# Tough and Stretchy: Mechanical Properties of the Alimentary Tract in a Fish Without a Stomach

**DOI:** 10.1093/iob/obac003

**Published:** 2022-02-08

**Authors:** Jaquan M Horton, John M Gosline, Emily Carrington

**Affiliations:** Department of Biology, University of Washington, Seattle, WA 98195, USA; Department of Zoology, University of British Columbia, V6T 1Z4 Vancouver, Canada; Department of Biology, University of Washington, Seattle, WA 98195, USA

## Abstract

The mechanical properties of intestinal tissues determine how a thin-walled structure exerts forces on food and absorbs the force of food as it enters and travels down the gut. These properties are critically important in durophagous and stomachless fish, which must resist the potential damage to foreign bodies (e.g., shells fragments) in their diet. We test the hypothesis that the mechanical properties of the alimentary tract will differ along its length. We predict that the proximal region of the gut should be the strongest and most extensible to handle the large influx of prey often associated with stomachless fish that lack a storage depot. We developed a custom inflation technique to measure the passive mechanical properties of the whole intestine of the stomachless shiner perch, *Cymatogaster aggregata*. We show that mechanical properties differ significantly along the length of the alimentary tract when inflated to structural failure, with 25–46% greater maximal stress, strain, extension ratio, and toughness at the proximal (25%) position. We also find that the alimentary tissues (excluding the heavily muscular rectum) are generally highly extensible and anisotropic, and do not differ in wall circumference or thickness along the alimentary tract. These findings contribute to our knowledge of the mechanical properties of fish intestinal tissues and guide future studies of factors influencing the evolution of fish alimentary systems.

## Résumé

Les propriétés mécaniques des tissus intestinaux déterminent la manière dont une structure à paroi mince exerce des forces sur les aliments et absorbe leur force lorsque ceux-ci pénètrent et descendent dans l'intestin. Ces propriétés sont d'une importance capitale chez les poissons durophages et ceux sans estomac qui doivent résister aux possibles dommages que peuvent provoquer l'ingestion de corps étrangers (comme des fragments de coquilles) lorsqu'ils s'alimentent. Nous testons l'hypothèse selon laquelle les propriétés mécaniques du tube digestif diffèrent sur sa longueur. Nous prédisons que la région proximale de l'intestin devrait être la plus solide et la plus extensible pour gérer le grand afflux de proies souvent associé aux poissons sans estomac qui n'ont pas de capacité de stockage. Nous avons élaboré une technique de gonflage propre pour mesurer les propriétés mécaniques passives de l'ensemble de l'intestin de la perche méné dépourvue d'estomac, *Cymatogaster aggregata*. Nous montrons que les propriétés mécaniques diffèrent significativement le long du tube digestif lorsqu'il est gonflé jusqu'à une défaillance structurelle, avec une contrainte maximale, une déformation, un rapport d'extension et une résistance supérieurs de 25 à 46% à la position proximale (25%). Nous constatons également que les tissus de l'appareil digestif (à l'exclusion du rectum fortement musclé) sont généralement très extensibles et anisotropes, et ne diffèrent pas par la circonférence ou l'épaisseur de la paroi le long du tube digestif. Ces résultats contribuent à notre connaissance des propriétés mécaniques des tissus intestinaux des poissons et orientent les futures études sur les facteurs influençant l'évolution des systèmes alimentaires des poissons.

## Introduction

Durophagous fish are predators that specialize in eating hard prey, generally by crushing the protective shells of mollusks and crustaceans (Nelson 2006). These fish generally process captured prey by fragmenting shells before they are ingested, or at times swallow intact prey items (Norton 1988); few regurgitate indigestible material (Hattin 1996; Hulsey 2006). This feeding ecology has been found in the fossil record dating back to the early Cambrian, with a marked increase in durophagy during the Mesozoic marine revolution (Oji et al. 2003; Zatoń and Salamon 2008).

Numerous studies have investigated the feeding mechanisms and performance of durophagous fish, focusing on bite force mechanics (Huber et al. 2005), cranial morphology (Dean et al. 2007), pharyngeal jaw structures (Grubich 2003), prey capture behavior and kinematics (Pietsch 1978; Sasko et al. 2006), and dentition specializations (Norton 1988). All of the previous studies examine aspects associated with mechanisms and processes that enable these fish to consume hard prey. Substantially less is known, however, about what happens after hard prey enter the digestive tract and more specifically, the effect of ingesting hard prey on intestinal tissue mechanics.

The alimentary system of fish is a dynamic system that performs a variety of functions, such as nutrient absorption (Cyrino et al. 2008), osmoregulation (Yuge et al. 2006), hormonal regulation (Taylor and Grosell 2006), immune defense (McGhee and Kiyono 1999), ion and water transport (Scott at al. 2006; Taylor and Grosell 2006), gas exchange (Wu and Chang 1945; Yadav and Singh 1980), and even defense (Brainerd 1994). These structures must also resist tissue degradation in the presence of digestive enzymes, such as lipase and trypsin (Sklan et al. 2004). In addition, if hard shell or exoskeletal fragments are ingested, as in most durophagous fish, then the intestinal tissues must also resist mechanical damage as indigestible material passes through this complex system.

Many teleost fish lack a true stomach (e.g., Cyprinidae, Cyprinodonitdae, Labridae), which potentially is a plesiomorphic or secondary neotenic character (Barrington 1957; Kobegenova 1988; Harada et al. 2003). A stomachless state has been hypothesized to be advantageous for its ability to provide an alkaline intestinal tract for fish both in freshwater environments (Williams and Eddy 1986), and those with calcium carbonate rich diets such as durophagous fish (Lobel 1981), as well as to reduce energetic costs (Day et al. 2011). Moreover, there are also known differences in the structural morphology of the alimentary tract, e.g., thickness, folding, stomach, and gut distention (Suyehiro 1942; Al-Hussaini 1949; Burnstock 1959; German et al. 2010). Despite the lack of distinct structural compartmentalization in stomachless fish, there must be greater proximal expansion of the gut in order to accommodate the initial influx of whole-food items, or bolus, as they enter and travel along the digestive tract. However, how the material properties and mechanical function change along the length of the alimentary tract it is not well understood. If the intestinal tissues do not sufficiently distend, large foreign bodies or prey items (e.g., shell fragments) exerting forces on this thin-walled structure, could lead to mechanical damage, which in turn could ultimately lead to death (Compton and Kerfoot 2004). Given the necessary expansion and body confinement of the alimentary tract, knowledge of the stress, strain, extensibility, and toughness of these tissues are needed to fully understand the evolutionary history and function of alimentary tissues.

The relative symmetry and agastric nature of the alimentary system of the shiner perch *Cymatogaster aggregata* (Gibbons 1854) offers an ideal system to investigate the mechanical properties of gut tissues. In this study, we test the hypothesis that the mechanical properties of the shiner perch alimentary tract differ along the length of the alimentary tract, with stronger, tougher, and more extensible values near the proximal end to handle high load forced in by swallowing. We develop an inflation technique to measure the passive mechanical properties of whole alimentary tissues in fish under quasi-static pressure testing to (1) quantify alimentary tissue stress, strain, toughness, and extension ratio at three relative spatial positions (25%, 50%, 75%) along the length of the tract; and (2) assess whether tissue properties at the three relative positions exhibit anisotropic characteristics (i.e., radial extension is different than longitudinal extension). This study establishes a new method that shows how the spatial variation in the mechanical properties of alimentary tissues in a stomachless and durophagous teleost fish can accommodate a complex and mechanically challenging diet.

## Materials and methods

### Specimens

**Fig. 1:**
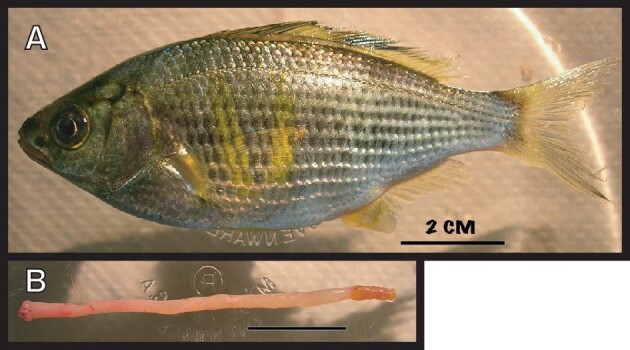
(A) Photograph of the durophagous and stomachless shiner perch, *Cymatogaster aggregata*. (B) Extracted and unfurled “S-shaped” alimentary tract in the zero-stress state. The anterior portion begins just distal to the pharyngeal apparatus and posterior segment ends just proximal to the cloaca; scale bar = 2 cm.

The shiner perch, *Cymatogaster aggregata* (Gibbons 1854) is a stomachless, viviparous, and demersal fish that inhabits coastal areas rich in eelgrass beds along the eastern Pacific from Baja, Mexico to Wrangell, Alaska (Eschmeyer et al. 1983; Fig. 1A). Twenty *C. aggregata* (82–96 mm SL; 11.4–17.9 g) were collected by seine at Jackson Beach, San Juan Island, Washington, USA (Lat 48°31’12’’N; long 123°00’36’’W). Fish were maintained in flow-through seawater tanks (128 L × 76 W × 24 H cm) at Friday Harbor Laboratories (FHL), University of Washington, for a period of 4–6 days to eliminate preexisting digestive material. All animal experiments were performed in accordance with the University of Washington Institutional Animal Care and Use Committee (IACUC).

### Fish and Alimentary tissue preparation

Fish were euthanized with 350mg/L of buffered Tricaine Methanesulphonate (MS–222) in seawater. Fish body mass (*BM*) was weighed (± 0.1 g), and standard length (*SL*), gape height (*GH*), and gape width (*GW*) measurements were taken using digital calipers (± 0.1 mm). Gape area (*GA*, in mm^2^) was calculated using the area of an ellipse:

(1) \begin{equation*}GA\ = \ \pi *\left( {GH/2} \right)*\left( {GW/2} \right).\end{equation*}

Fish were then separated into two groups with similar morphological traits (i.e., total length and mass). Complete alimentary tracts were then meticulously dissected from all individuals and unfurled to avoid mechanical tissue damage (Fig. 1B). The total length of each alimentary tract was then measured and immediately placed in a teleost Ringer's solution at 12°C until the cessation of smooth muscle activity was observed (*approximately* 40 to 60 min); this procedure was repeated for all individuals in both groups. Upon removal from the Ringer's solution, the lengths of each alimentary tract were blot dried and marked at three relative positions (i.e., 25, 50, and 75%) with red recorder ink. Approximately five mm of tissue was trimmed from the proximal end of the alimentary tract (to the bile duct) and one cm from the distal rectal region (*RR*), which had a distinct brown color and firmness when handled. The alimentary tracts from the inflation group were marked again with red recorder ink at five mm increments to measure longitudinal expansion during materials testing. These tissues (approximately 83 mm in length) were then returned to Ringer's solution until the inflation test was performed. The alimentary tracts from the other group were sectioned into three segments of similar length and transferred for histological analyses.

### Histological preparation & analysis

Tissue segments designated for histological analyses via light microscopy were placed in Trump's fixative (4% formaldehyde, 1% glutaraldehyde in 10 mM monobasic sodium phosphate, and 6.75 mM sodium hydroxide) (McDowell and Trump 1976) for 24–32 h at 4°C; the solution was buffered to a pH of 7.5 to prevent degradation of tissue ultrastructure. Tissue segments were removed from the fixative and rinsed four times in 0.1 M Phosphate buffered saline (PBS) at pH 7.5 for 20 min and stored in PBS overnight at 4°C. Samples (approximately 23 mm in length) were then rinsed with DI water, dehydrated in a graded ethanol (EtOH) series, and stored in 70% EtOH. In preparation for paraffin embedding (Paraplast X-Tra, Oxford labware; cat#8889-503002), tissues were run through an additional ethanol series of 80%, 90%, and two changes of 100% EtOH for 45 min. Gut tissue segments were then placed in a 1:1 100% EtOH/Xylene solution for 45 min, transferred to Xylene for 45 min (twice), and moved into a 1:1 Xylene/Paraffin solution for 45 min. Segments were then infiltrated in three changes of paraffin for 45 min in a vacuum oven at 60°C. Tissues were transferred into an embedding mold with heated forceps, and the resulting paraffin blocks were stored at room temperature until sectioned.

**Fig. 2:**
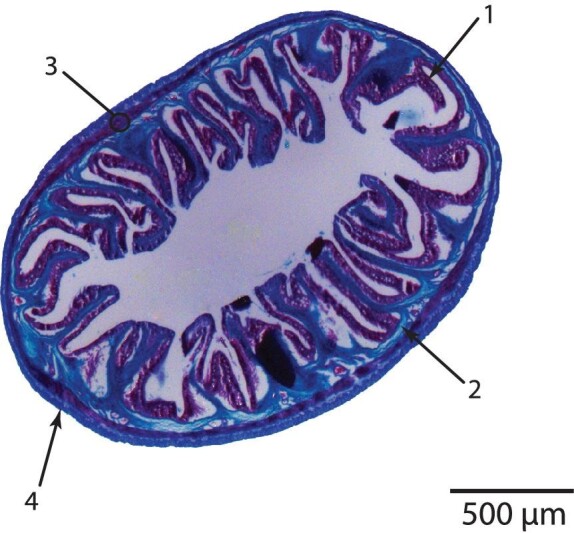
Representative radial cross-section showing the general wall structure of the alimentary system of the shiner perch, *Cymatogaster aggregata*, which consists of four layers: (1) tunica mucosa (mucosal epithelium and vascularized connective tissue), (2) submucosa (connective tissue), (3) tunica muscularis (muscle tissue), and (4) tunica serosa (mesothelial cells and vascularized connective tissue).

Paraffin blocks were trimmed to the midpoint of the tissue segment and then serially sectioned at 7 μm using a mechanical microtome (model 820, American Optical Co., Buffalo New York, USA). Sections were mounted on slides and stained with a modified Milligan's Trichrome for identification of muscle and collagen (Milligan 1946). Photographs of tissue cross-sections from three relative position segments (25%, 50%, and 75%) along the alimentary tract were taken with a Zeiss Axiocam HR camera (Axiovision Software) mounted on an Olympus SZX-12 microscope. Images were then analyzed in ImageJ (NIH v.1.46r) to identify gut perimeters and measure the perimeters of the outer and inner wall in order to quantify the wall radii, since fresh material for inflation tests could not be fixed or cut beforehand. The fixation process caused the length of the alimentary tract to contract between 7–10% from the original length. In anticipation of this shrinkage, we assumed a constant volume and used the actual outer diameter and relative percent wall thickness of histological segments for data analysis (Fig. 2; see *computational analysis* below).

### Inflation tests

**Fig. 3:**
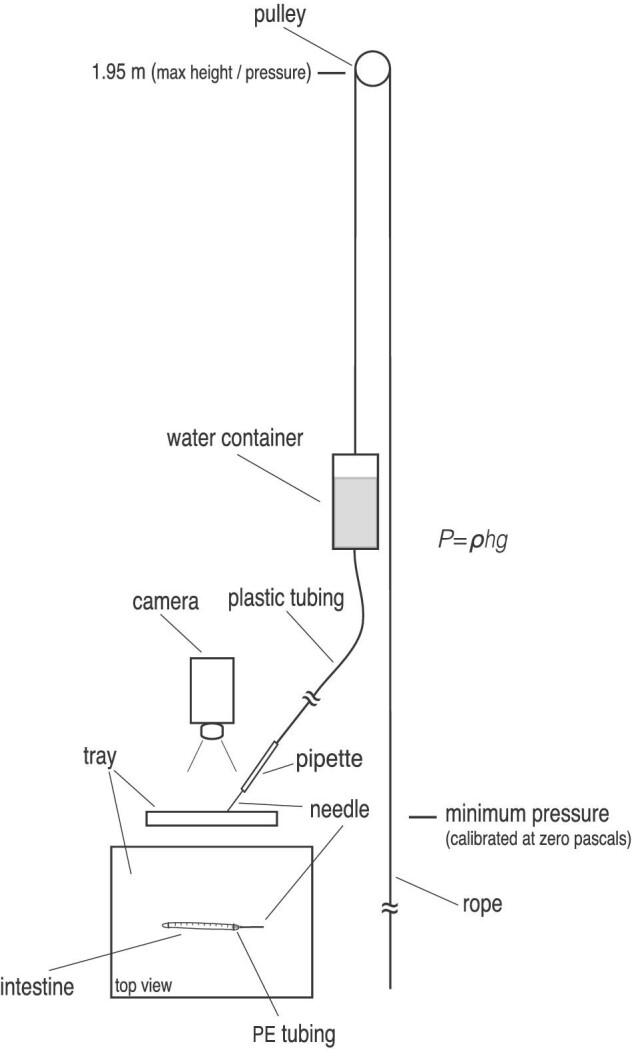
Schematic of the pressure device used for inflation tests; see detailed description in the methods section. The break in the illustrated plastic tubing and rope indicates length changes of the material depending on height of water container, and the tray containing the intestinal segment is shown in side and top view. Applied pressures ranged from 0 to 18,100 Pa and caused gut inflations up to 8.8 mm from an initial diameter of approximately 2 mm.

A high-definition camera was mounted over the testing area to record the inflation behavior of a whole alimentary tract (Fig. 3). For the first group of fish, one end of an intestinal tract was sutured onto a two cm piece of polyethylene (PE) 40 tubing with human hair and sealed with a small drop of cyanoacrylate. The PE tubing was then affixed to a syringe linked to a custom pressure device (Fig. 3). Static pressure was determined using the equation *P* = *ρhg*, where *P* is the pressure, *ρ* is the density of seawater, *h* is the height the water container was raised, and *g* is the acceleration of gravity.

System pressure was slowly increased from 0 to 200–400 Pa to flush out excess lumen material and fill the tract with seawater. The pressure was then lowered back to a resting pressure of zero before testing began. The distal end of the intestinal segment was then sealed shut with hair sutures and an additional drop of cyanoacrylate was added; segments were then submerged under 5 cm of seawater. The pressure of the system was increased by 200 Pa increments to 1000 Pa, and then increased by increments of 500 Pa thereafter. Pressure equilibrium was reached within the alimentary tract before each incremental increase, generally within 30–60 s, to allow for creep in the tissue. This procedure was repeated until structural failure of the whole intestine occurred.

### Data and computational analysis

For all individuals, still photographs from each incremental pressure during the test run were labeled and transferred to NIH ImageJ (v. 1.46r). The total length of the alimentary tract and the diameter at three relative positions along the length of the structure (25, 50, 75%) at each pressure were measured for all individuals.

We used the following equations to quantify the inner radius at a given relative position and pressure, assuming a constant alimentary wall volume (the wall thins as it expands) and circular in cross-section. First, constant volume (CV, in m^3^) was calculated as

(2) \begin{equation*}{\rm{CV\ }} = \ {L_o}*\ \pi \ \left( {r_{outer,\ initial}^2 - r_{inner,initial}^2} \right),\end{equation*}

where *L_o_* is the initial length (in m) of the alimentary segment, and *r_outer, initial_* and *r_inner__, initial_* are the outer and inner radii (in m), respectively, of the intestinal lumen at zero pressure. The outer radius was measured at the relative position, and corresponding mean histological percent wall thickness (Equation 10) was used to find the inner radii. The total, wall, and lumen areas (in m^2^) were calculated as follows:

(3) \begin{equation*}{\rm{Are}}{{\rm{a}}_{total}} = \ \pi *\ r_{outer}^2,\end{equation*}

(4) \begin{equation*}{\rm{Are}}{{\rm{a}}_{wall}} = \ {\rm{CV\ /\ L,}}\end{equation*}

(5) \begin{equation*}{\rm{Are}}{{\rm{a}}_{lumen}} = \ {\rm{Are}}{{\rm{a}}_{total}} - \ {\rm{Are}}{{\rm{a}}_{wall}},\end{equation*}

where *L* is the instantaneous length of the specimen. At each pressure, the outer radius (*r_outer_*, half the alimentary tissue diameter, in m), inner radius (*r_inner_*), and mid-wall radius (*r_mid-wall_*) associated with alimentary tissue distension were determined by using the following formulae:

(6) \begin{equation*}{r_{inner}} = ({\rm{Are}}{{\rm{a}}_{lumen}}/\pi )^{\frac{1}{2}},\end{equation*}

(7) \begin{equation*}{r_{mid - wall}} = \left( {{r_{outer}} + \ {r_{inner}}} \right)/2.\end{equation*}

The thickness (*t*, in m) of the intestinal wall at a given pressure was the difference between the outer and inner radii:

(8) \begin{equation*}t\ = \ {r_{outer}} - \ {r_{inner.}}\end{equation*}

To assess the relative expansion, or extensibility, of the inner lumen at a given pressure, the extension ratio (λ_inner_) was calculated as the ratio of the inner radius to the initial inner radius:

(9) \begin{equation*}\lambda \ = {r_{inner}}\ /{r_{inner,initial}}.\end{equation*}

The inner radius was used for all extension ratio calculations because this size determines the maximum size of an object that can move through the system within a specific region. The initial cross-sectional wall thickness was measured from the mean histological cross-sections from similar pairs, and calculated as a relative percent (%) of the overall radius of the structure using the following:

(10) \begin{equation*} {\rm{Percent\ wall\ thickness\ (\% )\ = \ }}( {{t_{initial}}*100} )/{r_{inner}}\end{equation*}

There is a very small constant pressure differential acting between the outer and inner walls of thin-walled structures (as strain varies across the wall thickness), so the mid-wall radius was used to calculate the circumferential stress for each cross-sectional tissue segment at a given incremental pressure (*P*). Thus, using the mid-wall extension ratio in determining the material properties provides average properties for the wall of the alimentary tract segment. The circumferential, or engineering, hoop stress σ (in *Pascals*, derived using *Laplace's Law* for a closed cylinder; Vogel 2003, 51) was calculated as

(11) \begin{equation*}\sigma = P*r_{mid - wall}/t,\end{equation*}

whereas strain, ε, was determined from the mid-wall extension ratio:

(12) \begin{equation*}\varepsilon \ = \ {\lambda _{mid - wall}} - 1.\end{equation*}

**Fig. 4:**
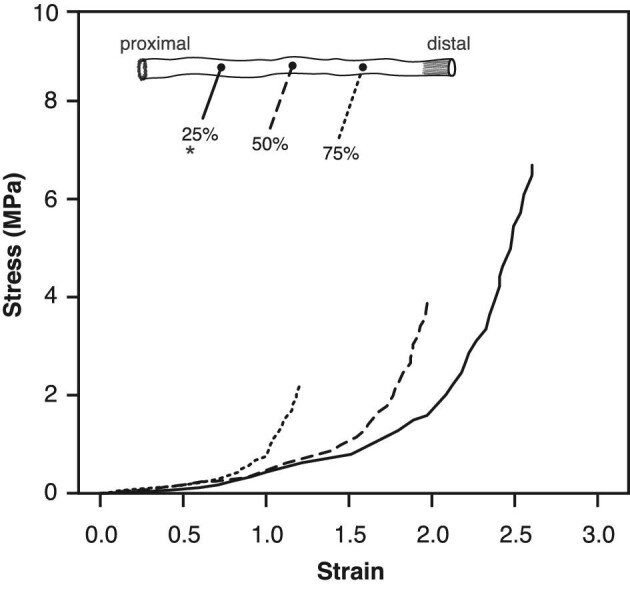
Representative stress-strain curve of the alimentary tract at three relative positions (25%–solid line, 50%–large dash, 75%–small dash) when inflated to structural failure. Curves exhibit the typical non-linear J-shaped stress-strain relationship of many pliant biological materials. The alimentary tissues are highly extensible under low pressures and then become increasingly stiff with increasing pressure, and the 25% position is overall more extensible compared to the two more distal positions. Asterisk denotes the location where failure always occurred, at the 25% position, providing an estimate of the ultimate material properties (i.e., breaking stress, breaking strain). Tissue from the 50% and 75% positions remained intact, which may underestimate ultimate material properties at these locales.

From stress versus strain relationships (Fig. 4), we quantified several alimentary mechanical properties at each position at structural failure: maximal stress (MPa), maximal strain (ε), toughness (MJ/m^3^) as the area under the curve that represents the energy the tissue can absorb before structural failure occurred, and the maximal extension ratio or extensibility (λ) as the maximal radial extension of the gut relative to the initial no-load state. Maximal radial extension provides a rough estimate of the maximal bolus or prey size that can be passed down the alimentary tract.

We calculated the circumferential to longitudinal strain ratio of each pressure trace to determine whether alimentary material conformed to *Laplace's Law*, which states that in a simple closed cylinder the hoop stress is twice the longitudinal stress, i.e., radial growth is twice the increase in length. Circumferential strain was found to be substantially higher, so we focused our statistical analysis on circumferential expansion for this study.

### Statistical analyses

Data were analyzed using the statistical program R (v. 2.15.3 GUI 1.53; R Foundation for Statistical Computing, Vienna, Austria). Fish of similar lengths were used, and data were normally distributed. A Student's *t*-test was used to evaluate morphological differences between the two fish groups. An analysis of variance (ANOVA) was used to evaluate the effect of relative position (25, 50, and 75%) along the length of the alimentary tract on each mechanical property (i.e., stress, strain, toughness, strain ratio). If the effect of relative position was significant, a post-hoc Tukey HSD test was used to compare between pairs of positions.

## Results

### Fish and alimentary morphometrics

**Fig. 5:**
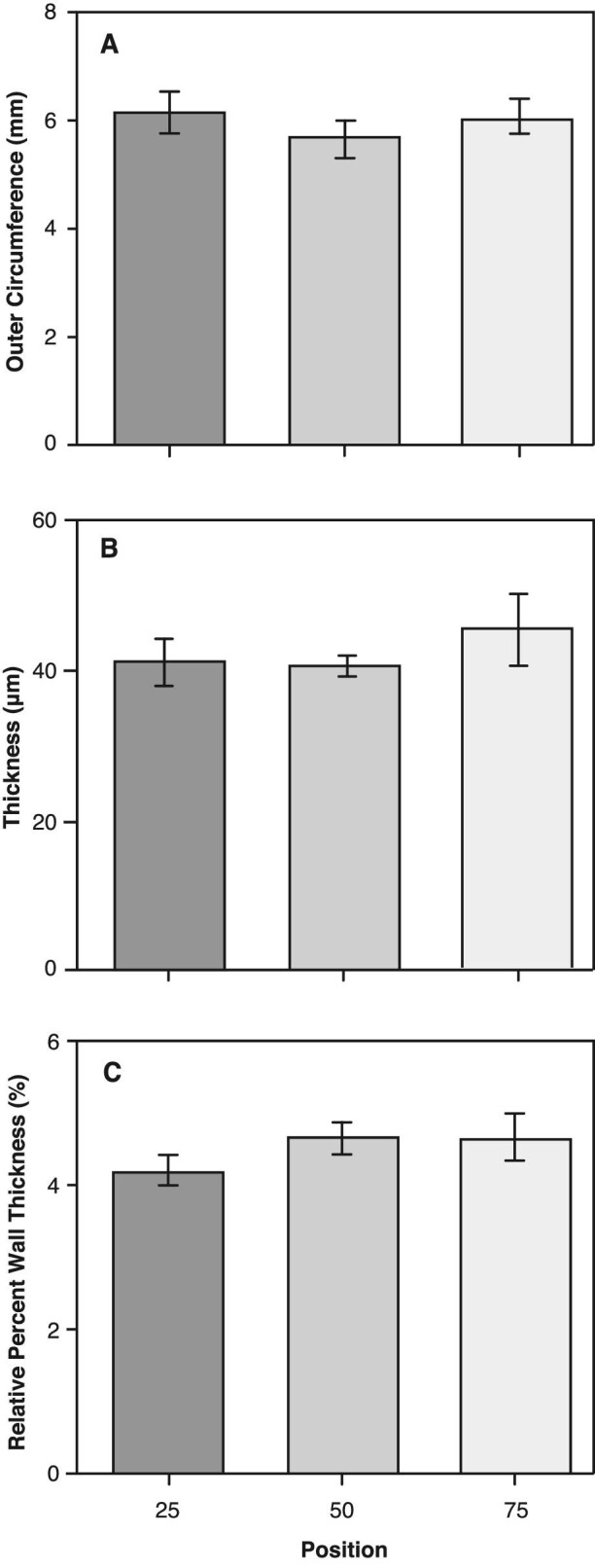
Outer radius (A), wall thickness (B), and relative radial percent thickness (C) at three relative positions (25, 50, and 75%) along the length of the alimentary tract, measured from histological cross-sections. Bars are means ± SE, n = 10 for each position. There were no significant differences among groups (Tukey HSD, *P*<0.05).

All morphometric measurements are listed in Table 1. The length range of the whole alimentary tract for the fish used in the inflation and histology analyses were 69–94 and 73–91 mm, respectively. There were no significant differences in any of morphological metrics between the two analyses groups (P = 0.33–0.87; Table 1). There was a distinct rectal region (RR) in each tract that was typically 13% of the total intestinal length. Additionally, there was no significant difference in the mean outer radius, wall thickness, or mean percent wall thickness among the three relative positions (25, 50, 75%) along the alimentary tract (*P* = 0.39–0.62; Fig. 5, Table 2); a similar finding by Young and Fox (1936). Overall, the load-bearing gut wall was approximately 5% of the structural radius.

**Table 1:** Summary of morphometric measurements of fish samples used for either inflation or histological analyses. Values are mean (± SE) and range; n = 10 for each analysis group. A Student's *t*-test was used to evaluate differences in morphology between the two analysis groups.

Analysis	Standard length (SL, mm)	Body mass (BM, g)	Gut Length (GL, mm)	Gape Height (GW, mm)	Gape Width (GW, mm)	Gape Area (GA, mm^2^)
Inflation						
mean + SE	86.9 ± 1.5	13.6 ± 0.6	83.3 ± 2.8	7.0 ± 0.2	5.1 ± 0.1	28.3 ± 0.8
range	81.5 − 96.7	11.4 − 17.9	69.2 − 94.3	6.0 − 8.0	4.9 − 5.5	23.6 – 32.4
Histology						
mean + SE	85.5 ± 1.0	13.4 ± 0.3	82.4 ± 1.8	7.0 ± 0.2	5.3 ± 0.1	28.9 ± 1.2
range	80.6 − 93.3	12.2 − 14.5	73.0 − 91.0	6.0 − 7.8	5.0 − 5.5	23.6 – 33.7
Student's *t*-test *t* (df = 18)						
*p-value*	0.865	0.700	0.738	0.760	0.339	0.779

**Table 2:** Summary of statistical analyses of histological parameters at three relative positions along the length of the alimentary tract. Values are mean (± SE) and range; n = 10 for each position. There was no significant difference among the three (25, 50, and 75%) relative positions. Overall, the load-bearing gut wall is roughly 5% of the structural radius.

	Mean ± SE for each position along length of alimentary tract			
Histological parameter	25%	50%	75%	ANOVA
outer radius (μm)	967.9 ± 58.4	889.5 ± 59.6	951.6 ± 60.5	*F*_2,27_ = 0.483; *P* = 0.622
wall thickness (μm)	41.1 ± 3.24	40.5 ± 0.15	45.6 ± 0.15	*F*_2,27_ = 0.578; *P* = 0.568
percent wall thickness (%)	4.25 ± 0.22	4.67 ± 0.24	4.71 ± 0.32	*F*_2,27_ = 0.970; *P* = 0.392

### Alimentary mechanical properties

**Fig. 6:**
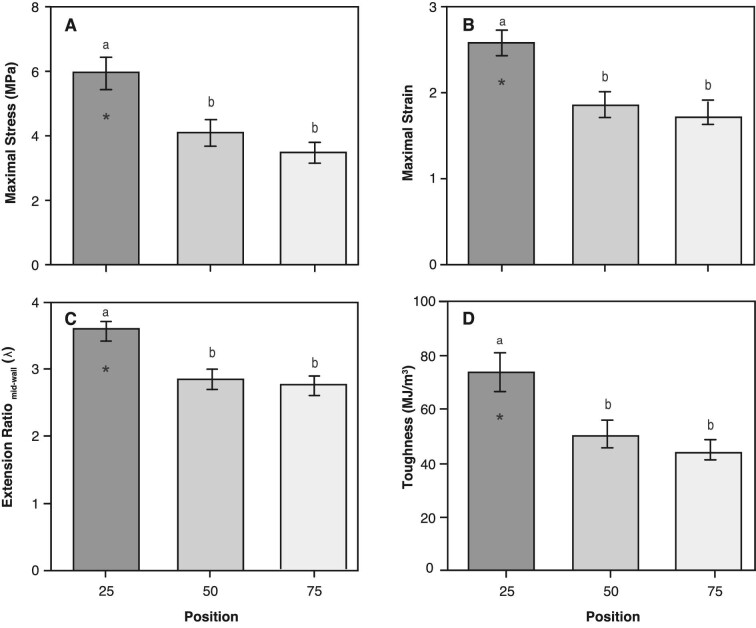
Summary of mechanical properties at three relative positions (25%, 50%, 75%) along the length of the alimentary tract when inflated to structural failure. All values are mean ± SE, n = 10 for each position. (A) Maximal stress, (B) maximal strain, (C) mid-wall extension ratio (λ), and (D) toughness. Different letter above bars indicate signicant differences among group (Tukey HSD, P<0.05). Asterisk denotes ultimate value where structural failure occurred (25% position) from maximal values where the tissue remained intact (50% and 75% positions).

The mechanical properties along the length of the alimentary tract at three relative positions (25, 50, and 75) were assumed to be independent of fish length over the small range of body sizes tested (Table 1). Tissue failure of the whole intestinal tract always occurred within the region of the 25% position. Therefore, values for ultimate mechanical properties refer to the 25% position, whereas values at the 50 and 75% positions correspond to maximal values measured at the point of structural failure. At structure failure, the maximal stress depended significantly on relative position and exhibited a decreasing trend distally (*F*_2,27_ = 8.634; *P*<0.01; Fig. 6A). Relative to the 50% locale, the more proximal 25% locale had significantly higher stress (by 46%) while the more distal 75% locale was not significantly different (Table 3).

**Table 3:** Summary of statistical analyses of the maximal mechanical properties at three relative positions along the length of the alimentary tract, and percent change relative to the midpoint (50%) position (n = 10 for each position) when pressurized to structural failure. Mean (± SE) values are shown for each mechanical property. Only the 25% position differed significantly from midpoint (50%) and distal (75%) positions (ANOVA, P< 0.01; Tukey HSD, *P* <0.05), and was a measure of the ultimate mechanical property; bold font). There was a significant difference in strain-ratio among the three relative positions and all values were considerably greater than the null strain-ratio of 2 for the inflation of a standard isotropic cylinder.

	Mean ± SE at each position along length of alimentary tract				Percent change relative to midpoint position		
Mechanical Property	25%	50%	75%	ANOVA	25%	50%	75%
Stress (MPa)	**5.92±0.50**	4.07±0.42	3.58±0.31	*F*_2,27_ = 8.634; *P*<0.01	**↑** 45.4%	-	**↓** 13.7%
Strain (ε)	**2.59±0.14**	1.87±0.15	1.77±0.15	*F*_2,27_ = 9.426; *P*<0.01	**↑** 38.4%	-	**↓** 5.3%
Extension ratio (λ)	**3.59±0.14**	2.87±0.15	2.77±0.15	*F*_2,27_ = 9.426; *P*<0.01	**↑** 25.0%	-	**↓** 3.3%
Toughness (MJ/m^3^)	**73.36±7.05**	50.58±4.89	44.70±3.39	*F*_2,27_ = 8.097; *P*<0.01	**↑** 45.0%	-	**↓** 11.6%
Strain ratio	**7.93±0.71**	5.83±0.72	5.74±0.92	*F*_2,27_ = 8.731; *P*<0.01	**↑** 36.0%	-	**↓** 1.5%

A similar pattern was observed in all of the other material properties we measured (Table 3). Mean maximal strain decreased significantly along the alimentary tract (*P* <0.01; Fig. 6B). Relative to the gut midpoint (50% locale), the more proximal 25% locale was 39% more extensible while the more distal 75% locale was not significantly different. Mean extension ratio (λ) decreased significantly along the alimentary tract (*P* <0.01; Fig. 6C). The gut at the proximal position (25% locale) was approximately 25% more extensible than at the midpoint (50% locale), and there was no significant difference in extension ratio between the 50 and 75% locales. This result translates into a total gut distension, from the initial no-stress state to maximal expansion, of approximately 360% percent in the proximal portion of the alimentary tract. The mean toughness (MJ/m^3^) also decreased significantly along the alimentary tract (*P* <0.01; Fig. 6D). Compared to the midpoint position (50%), the proximal position was 45.0% tougher, while the distal locale was not significantly different.

**Fig. 7:**
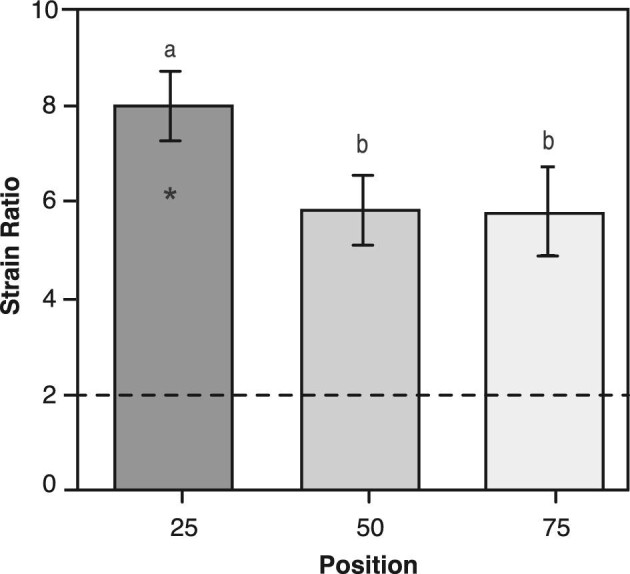
Strain ratio at three relative positions (25%, 50%, 75%) along the length of the alimentary tract at structural failure. All values are mean ± SE, n = 10 for each position. Asterisk denotes ultimate value where structural failure occurred (25% position) from maximal values where the tissue remained intact (50% and 75% positions). All values are substantially higher than the null strain ratio of 2 (dashed line) for a standard isotropic cylinder, which indicates the extended gut is a non-uniform anisotropic material that is reinforced in the longitudinal direction (Gosline 2018).

There was a significant difference in mean strain-ratio among the three relative positions of the alimentary tract (*P* <0.01, Table 3 and Fig. 7). Moreover, the mean strain ratios were 3.25 times higher than the null ratio of 2 for the inflation of a standard cylinder.

## Discussion

We developed a custom quasi-static pressure system to quantify the relative changes in the passive mechanical properties along the length of the alimentary tract of a stomachless fish, *C. aggregata*. These data support our hypothesis that the mechanical properties differ along the length of the alimentary tract. When inflated to structural failure, the maximal stress, strain, extension ratio, and toughness were significantly greater at the proximal (25%) position than the more distal (50 and 75%) positions along the alimentary tract (Table 3). In addition, the alimentary tissues were highly extensible and anisotropic (Fig. 6 and Fig. 7). Essentially, the initial 25% position expands and thins, absorbing more energy per unit tissue volume while withstanding a larger force per unit area (stress). This proximal area is important, as the influx of food exerts a force on the wall of the gut causing it to expand, and the ability of the gut to absorb this impact is critical to the gut not failing (i.e., rip, tear, rupture). We found no distinguishable difference in the initial wall thickness along the alimentary tract at the three relative positions (25, 50, 75%) (Fig. 5), suggesting these differences in mechanical properties are due to differences in the alimentary tissue structure and/or composition.

Durophagous fish differ in their approach in capturing prey items and few regurgitate indigestible material (Norton 1988; Hattin 1996; Hulsey 2006). Regardless of the potential evolutionary advantages associated with a lack of a “true” compartmentalized stomach, the alimentary tract must accommodate the initial influx of minimally processed hard prey items of durophagous fish. Al-Hussaini (1946) and Wallace et al. (2005) referred to the anterior portion of the intestinal tract of stomachless fish as an intestinal bulb that likely functions as a reserve. Our data are consistent with this hypothesis; we show the initial pressure associated with the influx of prey items effects a greater distension at the proximal (25%) region of the alimentary tract, indicating this position is capable of large-scale energy absorption and circumferential deformations (Fig. 6 and Table 3). This may also be the mechanism adopted by other stomachless fish to deal with the forces generated with primary ingestion (Sklan et al. 2004).

As discussed above, there were no significant differences in initial circumference or wall thickness at the three relative positions (Fig. 5A and B and Table 2). However, the mechanical properties differed significantly at the proximal, 25% position of the alimentary tract, with increases of 25–45% relative to the mid-section (Fig. 6, Table 3). These results suggest that the organization of the composite materials contribute to the differences in mechanical properties at the 25% position. The main structural components that maintain gut wall integrity are elastin and collagen, with smooth muscle providing minimal structural support (Krafka 1939). Elastin enables distension of the gut wall while the stiffer collagen fibers maintain structural integrity (Fung 1981; Wainwright et al. 1982; Storkholm et al. 1998). Therefore, changing the fiber orientation relative to the long axis of the structure could alter mechanical properties and cause anisotropy—where the properties differ depending on the direction of the applied load (Wainwright et al. 1978). In a closed, thin-walled pressurized cylinder, reinforcing fibers wound at 55° relative to the long-axis maintain its proportions and a strain ratio equal to 2, whereas fibers less than or greater than 55° produce shorter fat tubes or thinner elongate tubes, with strain ratios greater than or less than 2, respectively (Clark and Cowey 1958). Gosline (1971) showed that the circumferential elastic modulus in mesoglea is an order of magnitude greater than the longitudinal modulus due to an increase in reinforcing collagen fibers in the circumferential direction. While not investigated in this study, it is likely that variation in tissue ultrastructure and composition (i.e., collagen, elastin, smooth muscle) along the alimentary tract influences regional and anisotropic mechanical properties. This may be advantageous because the gut will experience differential stresses as non-uniform prey items, or bolus, decrease in size as they are processed and digested while travelling down the gut.

The extension ratio (λ) of the small intestine at full rupture was 3.6 in the shiner perch, substantially higher than in humans and guinea pigs (2.4 and 2.0, respectively, Storkholm et al. 1998; Egorov et al. 2002). The maximum extension ratio provides some guidance for estimating the maximal bolus size that can be ingested, which could ultimately dictate prey size and preference. Similarly, maximal gape area can serve as a proxy for the maximal cross-sectional area of prey a fish might ingest. This is a rough approximation, because presumably the pharyngeal jaws could assist in compressing the prey, and some materials and structures like fish fins, or segmented shells composed of chitin may be resistant to mechanical breakdown and simply compress and expand again once moved past the pharyngeal jaws (personal observation). If the gape area is smaller than the proximal gut area (a Gut-to-Gape Index, or GGI > 1), then the gut can safely accept prey and even allow for the expansion of prey once it enters the proximal part of the alimentary tract. However, if the gape area is larger than the proximal gut area (GGI <1), then some prey item packaging or processing would need to take place before entering the gut or moving down the alimentary tract in order to decrease the size and/or activity level of the prey item. The mean maximal gape area of the shiner perch in this study was 28.3 mm^2^, which corresponds to a GGI of 1.39 at the proximal position of the alimentary tract. This value is above the critical “safety factor” of 1, indicating the fish are likely not at risk for gut failure during the initial influx of prey items. In comparison, the GGI of the midpoint and distal positions was 0.62 and 0.45, respectively. These GGI values indicate the bolus must compress as food is broken-down and digested as it moves along the alimentary tract. In addition, the gut is a dynamic tissue so smooth muscle control, creep, or strain softening could be implemented if necessary to help resist tissue failure.

We pressurized whole intestinal tissues to maintain the interwoven cross-linking of the fibers throughout these structures (Orberg et al 1983; Gabella 1987). Thin-walled tubes are often cut into ring segments for testing properties (e.g., Lillie and Gosline 2007), which has the potential to destroy adjacent cross-links and destabilize the structural integrity of the tissue and alter the radial geometry. This damage and instability could lead to inaccurate measurement of tissue mechanical properties. A substantial limitation of our approach, however, is we can measure *ultimate* material properties only in positions where the tissue failed, in this case on the 25% position. Our comparison of the material properties along the alimentary tract reflect values obtained when the whole structure was inflated to failure, which potentially underestimates the ultimate material properties of the midpoint and distal positions. It is clear that although the pressure was the same throughout the tube, stress at the 25% position was highest, and the increased thinning of the tissue caused the structure to fail in this region. Clearly, the histology in this region is different than the 50% and 75% locations, causing the 25% region be more extensible and act like a “stomach,” which can accept the initial influx of prey material. However, a future study testing shorter relative segments (i.e., cut alimentary tract into thirds with the focal relative position in the center of the segment) may be warranted. This may determine if any adjacent histological structures at a given position, particularly connective tissues that run the length of the alimentary tract (e.g., submucosa), are providing indirect structural support to an upstream or downstream part of the alimentary tract. There is a possibility that tissue fibers run the entirety of the structure, which may add residual strength and influence the structural dynamics of these tissues. Puncture testing would be also be interesting, and mucus production and increased knowledge of prey diversity would be needed in order to determine an appropriate test method (e.g., probe size, probe shape, puncture rate). Knowledge of prey/bolus processing dynamics and its relation to tissue creep and stress relaxation could also be addressed in future studies. The findings of this study establish a framework for such tests of the distension limits of the alimentary tissues in fish.

Overall, this study used whole tissue inflation testing to establish that the mechanical properties differ along the length of the alimentary tract of a stomachless fish at tissue failure. To develop this method, we focused on a narrow size range of a single species. An interesting avenue for future studies would be to determine if the ultimate mechanical properties differ along the length of the intestinal tract, as well as whether mechanical properties differ during development, i.e., over an ontogenetic size range. Ferraris and Cruz (1987) have shown that histological characteristics are similar across developmental stages, yet the geometry of these regions change during development. In addition, ontogenesis leads to changes in intestinal length, prey preference, and trophic shifts in fish (Stoner and Livingston 1984; Kramer and Bryant 1995; Tibbetts and Carseldine 2005). There is therefore the potential for changes in mechanical properties across developmental stages. What remains to be determined is whether ontogenetic changes in alimentary tract morphology lead to associated changes in the mechanical properties of these structures, which will ultimately increase our understanding of the various selective pressures driving the evolution of intestinal tissues.

## Data Availability

Data available upon request to the authors.
